# Integrating Retrieval-Augmented Generation with Large Language Models in Nephrology: Advancing Practical Applications

**DOI:** 10.3390/medicina60030445

**Published:** 2024-03-08

**Authors:** Jing Miao, Charat Thongprayoon, Supawadee Suppadungsuk, Oscar A. Garcia Valencia, Wisit Cheungpasitporn

**Affiliations:** 1 Division of Nephrology and Hypertension, Department of Medicine, Mayo Clinic, Rochester, MN 55905, USA; miao.jing@mayo.edu (J.M.); charat.thongprayoon@gmail.com (C.T.); s.suppadungsuk@hotmail.com (S.S.); garciavalencia.oscar@mayo.edu (O.A.G.V.); 2 Chakri Naruebodindra Medical Institute, Faculty of Medicine Ramathibodi Hospital, Mahidol University, Samut Prakan 10540, Thailand

**Keywords:** large language models (LLMs), nephrology, chronic kidney disease, artificial intelligence, retrieval-augmented generation (RAG)

## Abstract

The integration of large language models (LLMs) into healthcare, particularly in nephrology, represents a significant advancement in applying advanced technology to patient care, medical research, and education. These advanced models have progressed from simple text processors to tools capable of deep language understanding, offering innovative ways to handle health-related data, thus improving medical practice efficiency and effectiveness. A significant challenge in medical applications of LLMs is their imperfect accuracy and/or tendency to produce hallucinations—outputs that are factually incorrect or irrelevant. This issue is particularly critical in healthcare, where precision is essential, as inaccuracies can undermine the reliability of these models in crucial decision-making processes. To overcome these challenges, various strategies have been developed. One such strategy is prompt engineering, like the chain-of-thought approach, which directs LLMs towards more accurate responses by breaking down the problem into intermediate steps or reasoning sequences. Another one is the retrieval-augmented generation (RAG) strategy, which helps address hallucinations by integrating external data, enhancing output accuracy and relevance. Hence, RAG is favored for tasks requiring up-to-date, comprehensive information, such as in clinical decision making or educational applications. In this article, we showcase the creation of a specialized ChatGPT model integrated with a RAG system, tailored to align with the KDIGO 2023 guidelines for chronic kidney disease. This example demonstrates its potential in providing specialized, accurate medical advice, marking a step towards more reliable and efficient nephrology practices.

## 1. Introduction

Large language models (LLMs) are a sophisticated type of artificial intelligence (AI), specifically designed for understanding and generating human-like language, thereby earning the alternative designation of chatbots. These models, like the generative pre-trained transformer (GPT) series, are trained on vast datasets of text and can generate text that is often indistinguishable from that written by humans. They can answer questions, write essays, generate creative content, and even write code, based on the patterns they have learned from the data they were trained on. The capabilities of LLMs continue to expand, making them a crucial and transformative technology in the field of AI [1]. ChatGPT, a prominent generative LLM developed by OpenAI, was released towards the close of 2022 [2]. The most recent version, GPT-4, is equipped with advanced capabilities in both text and image analysis and is collectively referred to as GPT-4 Vision [3]. Alongside ChatGPT, the current landscape of widely utilized LLMs encompasses Google’s Bard AI [4] and Microsoft’s Bing Chat [5]. These technological advancements have enabled their adoption and implementation across various domains, including business, academic institutions, and healthcare fields [1]. 

Within the healthcare sector, the evolving influence of AI is reshaping traditional practices [6]. Tools like AI-driven LLMs possess a significant potential to enhance multiple aspects of healthcare, encompassing patient management, medical research, and educational methodologies [7,8,9,10,11]. For example, some investigators have shown that they can offer tailored medical guidance [12], distribute educational resources [7], and improve the quality of medical training [7,13,14]. These tools can also support clinical decision making [15,16,17], help identify urgent medical situations [18], and respond to patient inquiries with understanding and empathy [19,20,21]. Extensive research has shown that ChatGPT, particularly its most recent version GPT-4, excels across various standardized tests. This includes the United States Medical Licensing Examination [22,23,24,25]; medical licensing tests from different countries [26,27,28,29,30]; and exams related to specific fields such as psychiatry [31], nursing [32], dentistry [33], pathology [34], pharmacy [35], urology [36], gastroenterology [37], parasitology [38], and ophthalmology [39]. Additionally, there is evidence of ChatGPT’s ability to create discharge summaries and operative reports [40,41], record patient histories of present illness [42], and enhance the documentation process for informed consent [43], although its effectiveness requires further improvement. Within the specific scope of our research in nephrology, we have explored the use of chatbots in various areas such as innovating personalized patient care, critical care in nephrology, and kidney transplant management [44], as well as dietary guidance for renal patients [45,46] and addressing nephrology-related questions [47]. 

Despite these advancements, LLMs face notable challenges. A primary concern is their tendency to generate hallucinations—outputs that are either factually incorrect or not relevant to the context [48,49]. For instance, the references or citations generated by these chatbots are unreliable [50,51,52,53,54,55,56,57]. Our analysis of 610 nephrology-related references showed that only 62% of ChatGPT’s references existed. Meanwhile, 31% were completely fabricated, and 7% were partial or incomplete [58]. We also compared the relevance of ChatGPT, Bing Chat, and Bard AI in nephrology literature searches, with accuracy rates of only 38%, 30%, and 3%, respectively [59]. The occurrence of hallucinations during the literature searches, combined with the suboptimal accuracy in responding to nephrology inquiries [47] and correctly identifying oxalate, potassium, and phosphorus in diets [45,46], compromises the reliability or dependability of LLM outputs, raising significant concerns about their practical application. In critical areas like healthcare decision making, the impact of such inaccuracies is considerably heightened, highlighting the need for models that are more reliable and precise. 

To address these challenges, various strategies have been developed. One such strategy is prompt engineering, like the multiple-shot or chain-of-thought prompting techniques [60,61,62,63,64]. This approach involves structuring the input prompt to encourage the model to break down the problem into intermediate steps or reasoning sequences before arriving at a final answer. By explicitly asking the model to generate a step-by-step explanation or “thought process”, chain-of-thought prompting helps the model tackle multistep reasoning problems more effectively, potentially leading to more accurate and interpretable answers [65,66,67]. Although this approach has proven beneficial in several contexts, it is not without its limitations. Concerns like scalability and the risk of embedding biases present significant challenges, necessitating meticulous prompt engineering to maintain the model’s adaptability while safeguarding its efficiency. 

Another strategy to enhance LLMs’ ability is the retrieval-augmented generation (RAG) technique [68]. The primary advantage of the RAG approach is that it allows the LLM to access a vast external database of information, effectively extending its knowledge beyond what was available in its training data. This can significantly improve the model’s performance, especially in generating responses that require specific factual information or up-to-date knowledge. 

This review aims to explore the potential application of LLMs integrated with RAG in nephrology. This review also provides an analysis of the strengths and weaknesses of RAG. These observations are essential for appraising the potential of sophisticated AI models to drive notable advancements in healthcare sectors, where both precision and contemporary knowledge are of utmost importance, thus redefining the benchmarks for AI deployment in key domains. 

## 2. What Is the RAG System?

The RAG approach is a method used in natural language processing and machine learning that combines the strengths of retrieval-based and generative models to improve the quality of generated text [68,69]. This approach is particularly useful in tasks such as question answering, document summarization, and conversational agents. In the dynamic field of medicine, the unique capability of the RAG system to access external medical databases in real time allows the LLM to base its responses on the latest research, clinical guidelines, and drug information [70,71]. 

To generate more accurate and contextually relevant responses, the RAG approach combines the strengths of two components including the retrieval and generation components. The former component is responsible for fetching relevant information or documents from a large database or knowledge source provided to the LLMs. The retrieval is typically based on the input query or context, aiming to find content that is most likely to contain the information needed to generate an accurate response. The latter component takes the input prompt along with the retrieved documents or information from the retrieval component and generates a response. The generation component uses the context provided by the retrieved documents to inform its responses, making them more accurate, informative, and contextually relevant. 

The RAG approach is particularly beneficial in scenarios where the model needs to provide information that may not have been present in its training set or when the information is continually updated. By grounding the responses in factual data, the RAG approach effectively reduces the occurrence of inaccuracy or hallucinations. However, the success of RAG depends on the quality and timeliness of the external data sources, and integrating these sources introduces additional technical complexities. Complementing these approaches is the process of fine-tuning, which involves adapting a pre-trained model to specific tasks or domains. This enhances the model’s capacity to process certain types of queries or content, thereby improving its efficiency and specificity for certain domains. While this method improves the model’s performance in specific areas, it also poses the risk of over-fitting in certain datasets, potentially limiting its broader applicability and increasing the demands on training resources.

## 3. Current Research Regarding the Application of RAG in Medical Domain

A recent study experimentally developed a liver disease-focused LLM model named LiVersa, incorporating the RAG approach with 30 guidelines from the American Association for the Study of Liver Diseases. This integration was intended to enhance LiVersa’s functionality. In the study, LiVersa accurately answered all 10 questions related to hepatitis B virus treatment and hepatocellular carcinoma surveillance. However, the explanations provided for three of these cases were not entirely accurate [72]. Another study introduced Almanac, an LLM framework enhanced with RAG functions, which was specifically integrated with medical guidelines and treatment recommendations [73]. This framework’s effectiveness was evaluated using a new dataset comprising 130 clinical scenarios. In terms of accuracy, Almanac outperformed ChatGPT by an average of 18% across various medical specialties. The most notable improvement was seen in cardiology, where Almanac achieved 91% accuracy compared to ChatGPT’s 69% [73]. Moreover, they evaluated the performance of Almanac against conventional LLMs (ChatGPT-4 [May 24, 2023 version], BingChat [June 28, 2023], and Bard AI [June 28, 2023]) by testing the LLMs with a new dataset comprising 314 clinical questions across nine medical specialties. Almanac demonstrated notable enhancements in accuracy, comprehensiveness, user satisfaction, and resilience to adversarial inputs when compared to the standard LLMs [74]. A recent investigation introduced a RAG system named RECTIFIER (RAG-Enabled Clinical Trial Infrastructure for Inclusion Exclusion Review), assessing its efficacy against that of expert clinicians in a clinical trial screening [75]. The comparison revealed a high concordance between the responses from RECTIFIER and those from expert clinicians, with RECTIFIER’s accuracy spanning from 98% to 100% and the study staff’s accuracy from 92% to 100%. Notably, RECTIFIER outperformed the study staff in identifying the inclusion criterion of “symptomatic heart failure”, achieving an accuracy of 98% compared to 92%. In terms of eligibility determination, RECTIFIER exhibited a sensitivity of 92% and a specificity of 94%, whereas the study staff recorded a sensitivity of 90% and a specificity of 84%. These findings indicate that integrating a RAG system into GPT-4-based solutions could significantly enhance the efficiency and cost effectiveness of clinical trial screenings [75]. 

The RAG’s strengths lie in its access to current information and its ability to tailor relevance. By utilizing the most recent data, the likelihood of offering outdated or incorrect information is greatly reduced. However, this approach also presents several challenges. The effectiveness of RAG’s responses is heavily dependent on the quality and currency of the data sources it uses. Adding RAG to LLMs also introduces an extra layer of complexity, which can complicate implementation and ongoing management. Moreover, there is a risk of retrieval errors. Should the retrieval system malfunction or fetch incorrect information, it could result in inaccuracies in the output it generates. 

## 4. The Potential Applications of RAG in Nephrology

The RAG integration is also valuable in nephrology, where staying abreast of the latest developments is crucial. This integration of current, validated data from external sources significantly reduces the likelihood of the LLMs providing outdated or incorrect information.

### 4.1. Integrating Latest Research and Guidelines

The RAG approach has the unique capability to dynamically integrate the most recent findings from nephrology-related sources into the model’s outputs. This includes new research from nephrology journals, results from the latest clinical trials, or any updates in treatment guidelines. By doing so, the RAG approach ensures that LLMs are not only up-to-date but also highly relevant and accurate in the field of nephrology. For instance, consider a scenario where a nephrology specialist or an internist is seeking information about the latest management strategies for polycystic kidney disease (PKD). In such cases, the RAG can actively search for, retrieve, and incorporate information from the most recent guidelines and treatment protocols, such as the KDIGO 2023 clinical practice guideline for autosomal dominant polycystic kidney disease (ADPKD), and studies published in the PubMed database. This process involves not just accessing this information but also synthesizing it in a way that is coherent and directly applicable to the query at hand.

By utilizing RAG, the physician is thus provided with information that is not only current but is also directly relevant to their specific inquiry. This approach is especially valuable in a field like nephrology, where advancements in research and changes in treatment protocols can have a significant impact on patient care. The ability of RAG to provide the latest knowledge helps healthcare professionals stay informed and make well-founded decisions in their practice.

### 4.2. Case-Based Learning and Discussion

Employing RAG in educational settings can significantly enhance the learning process by incorporating detailed and real-life case studies into lectures, discussions, or interactive learning modules. This application of RAG is particularly useful in complex and dynamic fields like medicine. Take, for example, the education of medical students on the topic of complex electrolyte imbalances in chronic kidney disease (CKD). The RAG approach can be utilized to access and reference specific, real-world case reports or clinical scenarios relevant to this topic. By doing so, it can provide students with practical, tangible examples that illustrate the theoretical concepts they are learning. This not only aids in a deeper understanding of the subject matter but also helps students appreciate the real-world implications and applications of their knowledge. 

Moreover, RAG’s ability to retrieve the latest studies and reports ensures that the educational content is not only rich in practical examples but also current. This is especially vital in medical education, where staying abreast of the latest research and clinical practices is crucial. By integrating up-to-date case studies and scenarios, RAG can help create a more engaging and informative educational experience, preparing students for the challenges they will face in their medical careers. This approach can be extended to other complex medical topics, making learning more interactive, relevant, and evidence-based.

### 4.3. Multidisciplinary Approach

In situations where a multidisciplinary perspective is essential, RAG proves to be particularly valuable as it can draw upon a wide array of medical disciplines to offer a more comprehensive understanding. This capability is critical in treating conditions that intersect multiple areas of healthcare. Consider the case of a patient suffering from diabetic nephropathy, for instance. This condition, being at the crossroads of diabetes and kidney health, requires a nuanced understanding from several medical specialties. The RAG system can effectively consolidate relevant information from endocrinology, focusing on diabetes management strategies; from cardiology, addressing the cardiovascular risks associated with the condition; and from nephrology, providing insights into preserving renal function. 

By integrating this diverse information, the RAG system can greatly assist healthcare professionals in developing a holistic and multifaceted treatment plan. This approach ensures that all aspects of the patient’s condition are considered, leading to more effective and comprehensive patient care. Such an integrated approach is beneficial not just in diabetic nephropathy but in any complex medical condition where multiple body systems are affected or where various specialties need to collaborate for optimal patient management. The ability of RAG to seamlessly merge insights from different medical fields into a cohesive whole enhances its utility in planning and implementing effective treatment strategies. 

## 5. Creation of a CKD-Specific Knowledge Base for RAG

**Figure 1:**
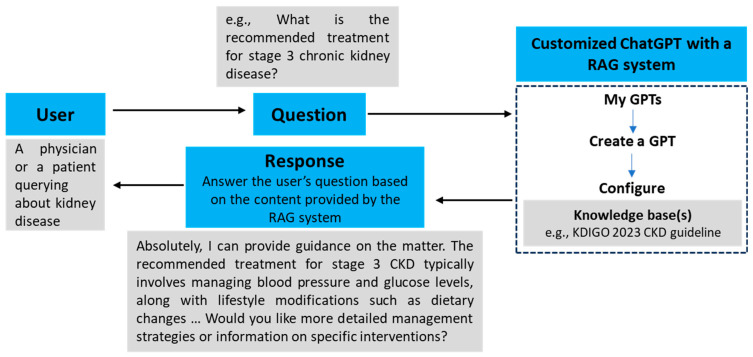
The process of creating a customized ChatGPT model with the retrieval-augmented generation (RAG) strategy in nephrology.

To illustrate the process of creating a customized ChatGPT model with a RAG strategy, we will use the field of nephrology as a reference, specifically focusing on CKD due to its prevalence in nephrology encounters (Figure 1). This example will serve to demonstrate the steps and considerations involved in tailoring a ChatGPT model to a specific medical specialty, incorporating a specialized knowledge base. The aim is to enhance the model’s responses with precise, specialized knowledge, in this case, centered around CKD, guided by insights from the KDIGO 2023 Clinical Practice Guideline [76]. Below is a detailed breakdown of the steps involved in this process. 

### 5.1. Creation of a CKD-Focused Retrieval System

This process involves the careful selection of knowledge sources, integration of guidelines, and regular updates to ensure accuracy and relevancy. The first step is to meticulously select a comprehensive database rich in information about CKD. This database should draw from a range of reliable sources, such as peer-reviewed academic journals, reports from clinical trials, and authoritative nephrology textbooks. A key focus is placed on incorporating the KDIGO 2023 CKD guidelines [76], which are recognized for their currency and authority in the field. 

**Figure 2:**
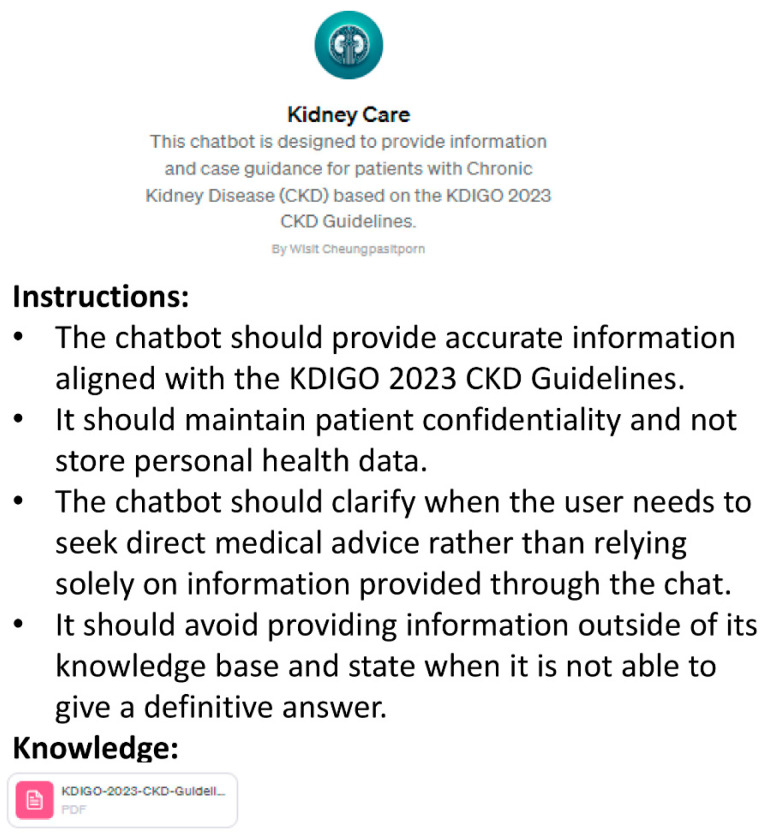
The creation of a CKD-specific knowledge base by customizing GPT-4 with the retrieval-augmented generation (RAG) approach.

Next, it is vital to directly integrate these KDIGO 2023 guidelines into the chosen database by creating a customized ChatGPT model (Figure 2). This process involves navigating to “My GPTs” and selecting “Create a GPT”. Following this, we have the opportunity to customize/configure our GPT by entering a name, description, and instructions, and by uploading the knowledge bases(s) we wish to embed within the model. We can choose to restrict access to the model by selecting one of the following options: “Only me”, “Anyone with a link”, or “Everyone”. Once customized, the GPT will be accessible under “My GPTs”, where it will produce responses utilizing the incorporated database(s).

This integration covers the detailed aspects of CKD, including diagnosis, staging, management, and treatment protocols. Such incorporation ensures that the model’s responses are in line with the most recent and accepted clinical practices. While ChatGPT operates based on its internal knowledge gained during training, RAG takes this a step further by dynamically incorporating external information into the generation process. The integration of a retrieval component in RAG could theoretically enhance ChatGPT by providing it access to a wider range of current information and specific data not covered during its training.

### 5.2. Development of a CKD-Focused Retrieval System

The RAG system, specialized for CKD, is specifically configured to identify and respond to CKD-related queries accurately. It is adept at grasping the intricacies of CKD, including its various stages, the comorbid conditions often accompanying it, and the diverse methods of treatment available. Additionally, the system is fine-tuned for both speed and relevance, ensuring rapid and efficient access to relevant information from the comprehensive CKD database when processing queries. This optimization guarantees prompt and pertinent responses tailored to the specifics of CKD. Moreover, establishing a system for continuous updates to the database is crucial. This involves regularly reviewing and including new research findings, updated medical guidelines, and emerging treatment methods in nephrology. Keeping the database up to date guarantees that the information remains both current and authoritative, making it a reliable foundation for the model’s knowledge base. 

### 5.3. Integration with the Customized GPT-4 Model

Integrating the customized GPT-4 model with the CKD retrieval system involves establishing strong and secure API (Application Programming Interface) connections. Firstly, it focuses on creating a robust connection that allows for the seamless flow of data between the customized ChatGPT model and the CKD retrieval system. This connection must be secure to protect sensitive medical information and ensure data integrity. Secondly, the customized ChatGPT model undergoes fine-tuning to harmonize the in-depth CKD information with its innate natural language processing abilities. This fine-tuning is critical to ensure that the model not only provides responses that are accurate and rich in CKD-specific information but also maintains clarity and appropriateness in the context of the user’s query. 

Through this integration, the model becomes capable of delivering responses that are not just factually correct but also tailored to the specific context of the query, whether it is a patient’s inquiry, a healthcare professional’s detailed question, or an educational scenario. This ensures that the model’s outputs are highly relevant, understandable, and useful for various users, ranging from medical practitioners and students to patients seeking information about CKD. 

### 5.4. Customized Response for CKD Inquiries

The integration of a customized GPT-4 model with a CKD-specialized RAG system brings a significant advancement in handling CKD-related inquiries. This integration leverages sophisticated algorithms to ensure that the ChatGPT model precisely recognizes the context and specific details of queries related to CKD, leading to highly relevant and tailored responses. This process operates on multiple levels, including contextual understanding, relevance of responses, access to updated information, and dynamic information integration. 

Through this integrated approach, the ChatGPT model becomes a powerful tool for providing accurate, up-to-date, and highly specific responses to a wide range of CKD-related inquiries. This capability is particularly valuable for healthcare professionals seeking quick and reliable information, patients looking for understandable explanations of their condition, and researchers needing the latest data in the field of nephrology. 

### 5.5. Rigorous Testing with CKD Scenarios

The system undergoes comprehensive testing in a variety of CKD situations. This testing encompasses a spectrum of patient histories, various stages of CKD, and the intricacies involved in treatment plans. Such extensive testing is crucial for confirming the model’s reproductivity and its ability to adapt to diverse clinical conditions. The feedback obtained from these rigorous tests is instrumental to the ongoing enhancement of the system. It aids in refining the precision of information retrieval and boosting the effectiveness of how the ChatGPT model works in conjunction with the CKD database. This process of continuous improvement ensures the system remains reliable and effective in addressing the complex needs of CKD management.

### 5.6. Regular System Monitoring and Updating

The system’s performance in providing accurate and relevant CKD information is consistently monitored. This includes assessing the accuracy of responses, the relevance of information provided, and the speed of retrieval. Moreover, the CKD database is regularly updated with the latest research, guidelines, and treatment protocols, ensuring the model’s responses remain current and authoritative. 

### 5.7. Healthcare Professional Engagement and Feedback

Healthcare professionals are trained on how to effectively use the customized ChatGPT model for CKD queries. This includes understanding its capabilities, limitations, and the best ways to phrase queries for optimal results. A feedback loop is established to continuously improve the system based on real-world user experiences and suggestions from healthcare professionals.

## 6. Examples of Responses Generated by GPT-4 with and without RAG System

**Figure 3:**
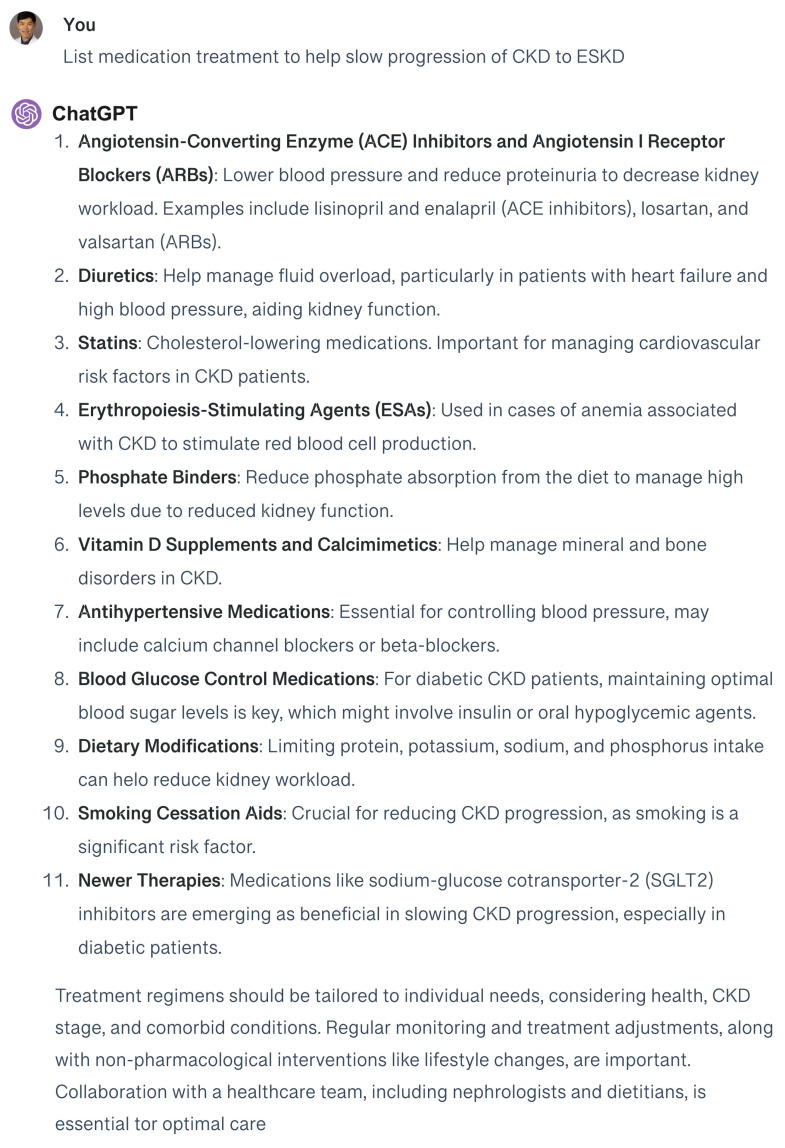
Responses of general GPT-4 to the question regarding medication treatment to help slow the progression of CKD to ESKD.

**Figure 4:**
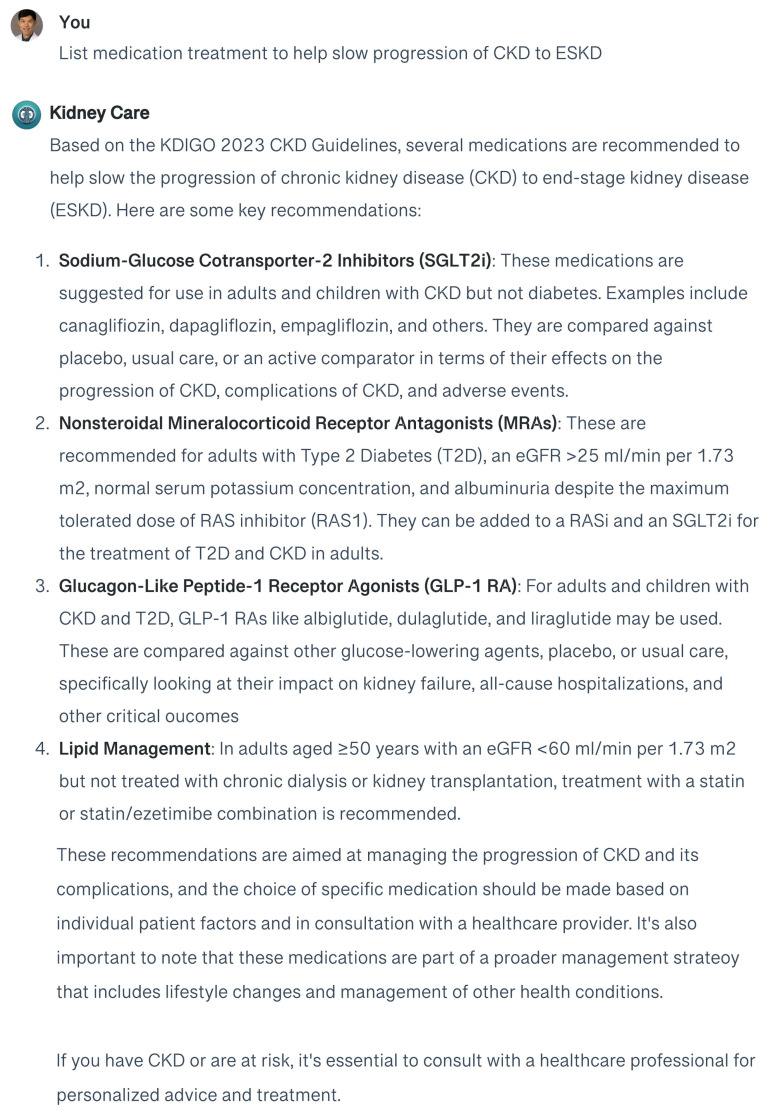
Responses of the customized GPT-4 with the retrieval-augmented generation (RAG) system to the question regarding medication treatment to help slow the progression of CKD to ESKD.

The effectiveness of the responses generated by GPT-4, both with and without the RAG approach, is evaluated using a straightforward query: “List medication treatment to help slow progression of CKD and end-stage kidney disease (ESKD)”. This test aims to compare the quality and accuracy of the information provided by GPT-4 under both methodologies (Figure 3 and Figure 4).

When using the general GPT-4 to address treatment approaches for slowing the progression of CKD to ESKD, the responses tend to offer a broad overview, lacking in-depth adherence to the latest KDIGO guidelines. However, the customized GPT-4 model enhanced with a RAG system provides responses that are more specific, detailed, and nuanced. Upon verification, these responses are found to be in close alignment with the KDIGO 2023 CKD guidelines, accurately reflecting the current research and clinical practices within nephrology. ChatGPT’s recommendations included SGLT-2 inhibitor and GLP-1 receptor agonists for patients with CKD and type 2 diabetes. However, ChatGPT failed to mention some targeted pharmaceutical interventions that may offer a way to slow CKD progression in individuals with specific causes, such as tolvaptan for ADPKD patients. To enhance its precision, it is necessary to incorporate additional resources, such as the ADPKD guidelines, into its reference database. This will enable ChatGPT to access a broader array of documents, facilitating the generation of more precise advice for CKD patients with specific conditions. 

Significantly, utilizing a series of prompts or exploring varied prompting techniques in standard ChatGPT, such as the chain-of-thought method and determining a specific CKD guideline for use, could also lead to more consistent responses with the RAG system. This review seeks to present an alternative strategy, the RAG system, for enhancing the effectiveness of LLMs and their applications, including in the context of CKD, to illustrate its utility. This method proves to be advantageous, efficient, and expedient when responses require dependence on specific or particular documents. Therefore, creating a customized ChatGPT model specifically for nephrology, with a focus on CKD and based on the KDIGO 2023 CKD guidelines, is an extensive and meticulous process. It involves building a specialized knowledge base, developing a dedicated retrieval system tailored to nephrology, and integrating this with the ChatGPT model. The process also includes fine-tuning the model to generate precise responses, conducting thorough testing to ensure reliability, continuously updating the system with the latest information, and maintaining engagement with healthcare professionals for feedback and validation. This development results in a model that stands out in offering specialized and accurate medical guidance for managing CKD. As such, it becomes an invaluable resource for healthcare providers, enhancing their ability to deliver informed and up-to-date care to patients with CKD. Notably, the ChatGPT model has merely presented an instance to illustrate the utility of the RAG approach. Additional research is required to confirm its dependability and enhance its efficacy in nephrology applications.

## 7. Future Studies in the Context of LLMs with RAG Systems in Nephrology

Future studies in the context of LLMs with RAG systems in nephrology are suggested to address several promising avenues. These could significantly enhance both the depth and breadth of nephrology research, clinical decision support, patient education, and personalized medicine.

Prospective studies would likely involve deploying RAG-enhanced LLMs in clinical settings as decision-support tools. Their effectiveness in assisting with real-time patient care decisions could be evaluated against traditional decision-making processes. Key metrics could include improvements in treatment time efficiency, accuracy in diagnosis, and patient satisfaction levels. Research could explore the seamless integration of LLMs with RAG systems into electronic health record (EHR) platforms, which is essential for enabling real-time, context-aware decision support for clinicians treating patients with kidney diseases. For instance, by leveraging the latest research findings, current guidelines, and patient-specific data, these models could assist in identifying subtle patterns or rare conditions that are difficult for humans to discern, thus improving the diagnostic accuracy for complex kidney diseases and tailoring treatment plans for individual patients with kidney diseases such as CKD or AKI. Future research might also explore automating the process of conducting systematic reviews and meta-analyses using LLMs with RAG systems. This could significantly speed up the synthesis of new research findings, ensuring that the nephrology practice remains at the cutting edge.

Moreover, the integration of nephrology-focused RAG systems with other medical domains could provide a more comprehensive patient care model. For instance, combining nephrology with cardiovascular data might better predict renal patients’ risk of heart disease. Studies could examine the outcomes of such integrations in improving the management of comorbid conditions. Combining insights from genomics, proteomics, and other omics technologies with LLMs and RAG systems also could lead to a more comprehensive understanding of kidney diseases and breakthroughs in precision medicine and novel therapeutic targets.

The development of adaptive learning modules using RAG-enhanced LLMs could offer personalized educational pathways for medical professionals. These modules could use real-time data to simulate patient scenarios, adapting to the learner’s responses and providing immediate feedback grounded in the latest clinical guidelines. To mitigate the risk of misinformation, future research might develop advanced fact-checking algorithms tailored to medical data nuances. These algorithms could cross-reference multiple authoritative databases before generating patient advice, ensuring a higher degree of accuracy in the information provided. The LLMs with a RAG system can also be utilized to provide personalized, easy-to-understand educational materials and support for patients with kidney diseases.

Furthermore, studies may explore the establishment of international consortia for the standardization of AI applications in nephrology. These networks could facilitate the sharing of best practices, the creation of diverse and comprehensive datasets, and the development of AI models that are generalizable across different populations and healthcare systems. This includes customizing models to account for genetic, environmental, and socioeconomic factors affecting kidney disease prevalence and treatment outcomes across different populations.

As LLMs with RAG systems rely on extensive data, future studies must address ethical and privacy concerns, ensuring patient data are used responsibly and securely. Therefore, research into the ethical implications of AI in nephrology will need to address consent processes for patient data, biases in AI training, and the transparency of AI decision-making processes. Regulatory studies might focus on developing frameworks for AI accountability and compliance with healthcare regulations like the Health Insurance Portability and Accountability Act (HIPAA). 

## 8. Conclusions

Combining LLMs with RAG systems in nephrology is a big step forward. It has the potential to change how we care for and educate patients in this specialized area. However, one of the main challenges is making sure the information they provide is accurate and reliable. To make these models better for their use in nephrology, strategies like using detailed prompting techniques, carefully applying RAG, and fine-tuning the models are important. As we move into this new phase, it is essential to have teams that include AI experts, kidney specialists, and ethicists. The goal is to improve AI so that it not only matches the skills of healthcare professionals but also adds to them. Achieving this is complex and very important. It requires a constant commitment to accuracy, innovation, and ethical practice. Through ongoing research, improvement, and a focus on patient welfare, we are getting closer to a future where AI plays a transformative role in healthcare, leading to better patient outcomes and more effective, knowledgeable healthcare systems. 

## Data Availability

Availability Statements are available in the original publication, reports, and preprints that were cited in the reference citation.

## Abbreviations

ADPKD: autosomal dominant polycystic kidney disease; AI: artificial intelligence; CKD: chronic kidney disease; EHRs: electronic health records; ESKD: end-stage kidney disease; GPT: generative pre-trained transformer; HIPAA: Health Insurance Portability and Accountability Act; LLMs: large language models; PKD: polycystic kidney disease; RAG: retrieval-augmented generation.
